# Draft Genome Sequence of Bacillus velezensis BZR 336g, a Plant Growth-Promoting Antifungal Biocontrol Agent Isolated from Winter Wheat

**DOI:** 10.1128/MRA.00450-20

**Published:** 2020-07-23

**Authors:** Alexey V. Garkovenko, Ilya Y. Vasilyev, Elena V. Ilnitskaya, Vitaly V. Radchenko, Anzhela M. Asaturova, Alexander E. Kozitsyn, Natalia S. Tomashevich, Alexander V. Milovanov, Tatiana V. Grigoreva, Margarita V. Shternshis

**Affiliations:** a Shemyakin-Ovchinnikov Institute of Bioorganic Chemistry of the Russian Academy of Sciences, Moscow, Russia; b Trubilin Kuban State Agrarian University, Krasnodar, Russia; c Kazan (Volga Region) Federal University, Kazan, Russia; d All-Russian Research Institute of Biological Plant Protection, Krasnodar, Russia; University of Arizona

## Abstract

Bacillus velezensis strain BZR 336g is a plant growth-promoting rhizobacterium isolated from a winter wheat rhizoplane from the Krasnodar region in Russia. In this study, we report the genome, including genes with known phenotypic function, i.e., the biosynthesis of secondary metabolites with fungicidal and plant growth-promoting activities. We sequenced and analyzed the complete BZR 336g genome using two different DNA preparation methods to help us better understand the origin of the antimicrobial and antifungal abilities and to weigh the biocontrol properties of this strain.

## ANNOUNCEMENT

Modern intensive agriculture is continuously being developed. There are many technologies that contribute to obtaining high yields of high-quality agricultural products; one of them is the use of effective biological products based on new microorganisms (1, 2). Plant growth-promoting rhizobacteria (PGPR) are a heterogeneous group of root-attached microbiota that are able to produce secondary metabolites such as phytohormones, to improve nutrient delivery from the environment to plant tissues, and to suppress phytopathogens, resulting in the stimulation of host growth and development and affecting farming yields (3).

Bacillus velezensis was previously described as an aerobic Gram-positive endosporogenous bacterium and a prospective PGPR (4); closely related strains, e.g., Bacillus amyloliquefaciens, have been known for root-attaching, biologically active compound-producing, pathogen-repressing, and plant growth-promoting abilities (5). The BZR 336g strain was originally isolated from a winter wheat rhizoplane, and the plant source was grown in the Krylovsky district of the Krasnodar region in Russia. The strain was described previously as Bacillus subtilis BZR 336g (according to phenotyping and 16S rRNA gene analysis data) (6) and was placed in the Departmental Collection of Useful Microorganisms for Agricultural Purposes (accession number RCAM01729) as a highly effective producer of hydrolytic protease and lipase ferments and as a suppressor of common crop phytopathogens, such as Fusarium graminearum, Fusarium culmorum, Microdochium nivale, and Pyrenophora tritici-repentis (7, 8). The BZR 336g strain has demonstrated commercial promise as a biological agent (9) for crop pest control (10).

A pure bacterial culture stored on nutrient agar (pancreatic hydrolysate of fish meal-peptone enzymatic digest-sodium chloride microbiological agar) at 5°C was obtained from the collection and reinoculated onto fresh nutrient agar. Subsequently, a single colony was picked using an inoculation loop for DNA extraction.

Whole-genome shotgun (WGS) DNA libraries were prepared in parallel using two different kits, the fragmentation-through-polymerization (FTP) kit (designed originally [11]) and the Nextera DNA Flex library preparation kit (Illumina, Inc., USA). The same genomic DNA extraction kit, i.e., the genomic DNA purification kit (Thermo Fisher Scientific, USA), was used for both kit preparations. After sequencing on the Illumina MiSeq platform, totals of 1,309,100 and 8,380,456 paired-end reads (with a median of 151 bp) were generated after quality filtration for the FTP and Nextera samples, respectively. The resulting genome assemblies consisted of 3,955,453 bp (FTP) and 3,923,596 bp (Nextera) in 289 and 38 (Nextera) contigs, respectively, with expected coverages of 49.69× (FTP) and 161.44× (Nextera) (using *B. velezensis* Hx05 [GenBank accession number CP029473] as a reference), *N*_50_ values of 400,785 (FTP) and 721,226 bp (Nextera), and G+C contents of 46.3% (FTP) and 46.4% (Nextera). The read preprocessing was performed using FastQC v.0.11.8, Trimmomatic v.0.39, and Cutadapt v.2.5, and the genomes were assembled with the SPAdes v.3.9.1 toolkit. All software was deployed in separate Docker containers. The genome assembly annotation was performed remotely with the NCBI Prokaryotic Genome Annotation Pipeline (PGAP) using the best-placed reference protein set and GeneMarkS-2+ annotation software v.4.10.

The BZR 336g WGS sequences for the FTP and Nextera samples contained 3,911 and 3,795 coding sequences, 467 and 454 hypothetical proteins, 16 and 8 rRNAs, and 133 and 109 tRNAs, respectively. The WGS processing data revealed strain-specific gene clusters responsible for antiphytopathogenic and plant growth-stimulating properties. Several biosynthetic gene determinants for lipopeptides (surfactin, bacillomycin D, fengycin, and bacillibactin) and polyketides (macrolactin, bacillaene, and difficidin) were found, confirming the strain’s ability to induce the phenotypic defense mechanism known as induced systemic resistance (12) to a broad spectrum of pathogenic microorganisms and viruses, as shown previously for B. velezensis G341 (13), B. velezensis ZJ20 (14), B. velezensis YJ11-1-4 (15), B. velezensis GQJK49 (16), B. velezensis 2A-2B (17), and B. velezensis FZB42 (18). There was also the *yxaL* gene variant, highly related to a gene of B. subtilis (GenBank accession number NC_000964.3), known as a cobaltochelatase and plant root development stimulator (19).

### Data availability.

The major project data-processing scripts are available at GitHub (https://github.com/ivasilyev/curated_projects/tree/master/vradchenko/lactobacillus_salivarius). The B. velezensis BZR 336g sequences obtained have been deposited in the NCBI databases under the accession numbers PRJNA588983 (BioProject), SRX8162225 (SRA), and WKLB00000000 (GenBank) for the FTP sequence and accession numbers PRJNA588983 (BioProject), SRX8162231 (SRA), and WKKU00000000 (GenBank) for the Nextera sequence.
